# ARHGEF10L Promotes Cervical Tumorigenesis via RhoA-Mediated Signaling

**DOI:** 10.1155/2021/6683264

**Published:** 2021-03-24

**Authors:** Junyi Tang, Kehua Fang, Chang Li, Xiaotian Chang

**Affiliations:** ^1^Medical Research Center of the Shandong Provincial Qianfoshan Hospital, Shandong University, Jinan, Shandong 250014, China; ^2^Department of Clinical Laboratory Medicine, The First Affiliated Hospital of Shandong First Medical University & Shandong Provincial Qianfoshan Hospital, Shandong Medicine and Health Key Laboratory of Laboratory Medicine, Jinan, Shandong 250014, China; ^3^Clinical Laboratory of the Affiliated Hospital of Qingdao University, Qingdao, Shandong 266000, China; ^4^Medical Research Center of the Affiliated Hospital of Qingdao University, Qingdao, Shandong 266000, China; ^5^Qingdao Municipal Engineering Technology Center for Bio-Markers of Major Diseases, Qingdao, Shandong 266000, China

## Abstract

**Background:**

Rho guanine nucleotide exchange factor 10-like protein (ARHGEF10L) is a member of the guanine nucleotide exchange factor family, which regulates Rho GTPase activities, thus contributing to tumorigenesis. Our previous study demonstrated a strong association between the ARHGEF10L gene and the risk of cervical carcinoma. This study investigated the pathogenic role and mechanism of ARHGEF10L in cervical tumors.

**Methods:**

The HeLa cell line, which was derived from cervical carcinoma, was transfected with ARHGEF10L-overexpressing plasmids or anti-ARHGEF10L siRNA. Cell counting kit-8 assays, wound-healing assays, and cell apoptosis assays were performed to investigate the effects of ARHGEF10L on cell activities. A Rho pull-down assay and RNA-sequencing analysis were performed to investigate the pathogenic pathway of ARHGEF10L involvement in cervical tumors.

**Results:**

ARHGEF10L overexpression promoted cell proliferation and migration, reduced cell apoptosis, and induced epithelial-to-mesenchymal transition (EMT) via downregulation of E-cadherin and upregulation of N-cadherin and Slug in transfected HeLa cells. The overexpression of ARHGEF10L also upregulated GTP-RhoA, ROCK1, and phospho-ezrin/radixin/moesin (ERM) expression in HeLa cells. RNA-sequencing analysis detected altered transcription of 31 genes in HeLa cells with ARHGEF10L overexpression. Kyoto Encyclopedia of Genes and Genomes (KEGG) and gene ontology (GO) pathway analyses identified significant differences in cyclin-dependent protein serine/threonine kinase activity, cell responses to vitamin A, and Toll-like receptor signaling pathways. Both real-time PCR and Western blotting verified the increased expression of heat shock 70 kDa protein 6 (HSPA6) in ARHGEF10L-overexpressing HeLa cells. Since we reported that ARHGEF10L played a role through RhoA-ROCK1-ERM signaling, an important pathway in tumorigenesis, and stimulated EMT and HSPA6 expression in liver tumors and gastric tumor cells, we suggest that ARHGEF10L is a novel oncogene in many tumors.

## 1. Introduction

Rho GTPases are a family of approximately 20 small G proteins that are key regulators of diverse cellular functions, such as cell growth, survival, motility, morphogenesis, and differentiation [1]. The most well-characterized members are Cdc42, Rac1, and RhoA. The active GTP-bound state and the inactive GDP-bound state are the two different Rho GTPase conformations. Proteins in the GTP-bound conformation can interact with different effector proteins to mediate the downstream signaling of Rho GTPases [2, 3]. Rho guanine nucleotide exchange factors (RhoGEFs) facilitate the exchange of GDP for GTP, which causes GTPase activation. RhoGEFs contribute to oncogenesis, hereditary disorders, and infectious diseases [4].

Rho guanine nucleotide exchange factor 10-like protein (ARHGEF10L), also named GrinchGEF, is a member of the RhoGEF family that plays a vital role in activating Rho GTPases. The structure of ARHGEF10L has the typical Dbl homology domain, a putative WD40-like domain, and two predicted transmembrane helices [5]. In a genome-wide single nucleotide polymorphism (SNP) association study, Stacey et al. showed that the SNP rs7538876 conferred a risk of cutaneous basal cell carcinoma and that the region surrounding rs7538876 contains the encoding ARHGEF10L [6]. Earp et al. showed that SNPs rs2256787 and rs10788679 in the ARHGEF10L gene were closely associated with epithelial ovarian cancer [7]. Recently, we used the Illumina GoldenGate Assay, Sequenom MassARRAY, and TaqMan polymerase to analyze the possible correlations between tag SNPs in the ARHGEF10L locus and various tumor risks. The genotyping analyses demonstrated a strong association of rs2244444 and rs12732894 in ARHGEF10L with liver cancer. That study also detected increased expression of ARHGEF10L in hepatocellular carcinoma tissues and demonstrated that this increased expression stimulates hepatocellular carcinoma cell proliferation and migration by activating the RhoA-ROCK1-phospho-ERM pathway and epithelial-to-mesenchymal transition (EMT) in two liver tumor cell lines and in tumor-bearing mice. This finding suggests that increased expression of ARHGEF10L plays an important role in hepatocellular tumorigenesis [8]. Furthermore, this study demonstrated that rs10788678, rs10888045, rs74059378, and rs12562076 had significant differences in allelic frequency and/or genotypic frequency between the cervical carcinoma cohort (*n* = 197) and the controls (*n* = 384) [8]. To verify the Illumina microarray genotyping results, seven SNPs within the ARHGEF10L locus were genotyped in an independent case-control study using Sequenom MassARRAY. The case-control analysis showed a significant difference in genotypic frequency for rs12067869 between the cervical carcinoma cohort (*n* = 258) and healthy controls (*n* = 760). Following multiple-test correction, these SNPs remained significantly different in their allelic frequency and genotypic frequency. The genotyping analysis suggests that the ARHGEF10L gene is also genetically correlated with the risk of cervical tumor risk. Thus, the present study aimed to investigate whether and how the ARHGEF10L gene is involved in cervical tumorigenesis.

## 2. Methods and Materials

### 2.1. Transfection of ARHGEF10L-Expressing Plasmids into HeLa Cells

The HeLa cervical carcinoma cell line was cultured in high-glucose Dulbecco's modified Eagle's medium (DMEM, Gibco, USA) supplemented with 10% fetal bovine serum (FBS, Gibco, USA) and 1% penicillin/streptomycin (Gibco, USA). Cells were cultured in a humidified atmosphere containing 5% CO_2_ at 37°C. HeLa cells (6 × 10^5^) were transfected with ARHGEF10L-encoding plasmids (2 *µ*g) using PolyJet^TM^ DNA In Vitro Transfection Reagent (SignaGen, USA) according to the manufacturer's protocol. The construction of the ARHGEF10L-expressing plasmids was described in a previous study [8]. The pcDNA 3.1 (+)-RFP expression vector was used as the control plasmid. At 48 h and 72 h after transfection, ARHGEF10L expression was analyzed using real-time PCR and Western blotting to verify the transfection efficiency. All experiments were repeated at least three times.

### 2.2. Transfection of Interfering siRNA into HeLa Cells

SiRNA oligonucleotides targeting the ARHGEF10 L gene (target sequence: 5′-CCGCGTGAAGGAGATCCTG CA-3′) were designed and synthesized by QIAGEN (Germany). Cultured HeLa cells (6 × 10^5^) were transfected with 150 ng of siRNA using HiPerFect Transfection Reagent (QIAGEN, Germany) according to the manufacturer's protocol. AllStars siRNA, which does not correspond to the target sequence in the human genome, was used as a control. ARHGEF10L expression was analyzed using real-time PCR and Western blotting to verify the transfection efficiency. All experiments were repeated at least three times.

### 2.3. Quantitative Real-Time Polymerase Chain Reaction (qRT-PCR)

After transfection, total RNA was extracted from HeLa cells using the RNAprep Pure Cell Kit (TIANGEN, China) and then reverse-transcribed into cDNA using the RNA PCR Kit (TOYOBO, Japan) according to the manufacturer's instructions. Real-time PCR was conducted using a ViiA7 DX Instrument (Life Technologies, USA). The reactions were completed in a total volume of 10 *µ*l containing 5 *µ*l of SYBR Green Real-Time PCR Master Mix (TOYOBO, Japan), 2 *µ*l of ddH_2_O, 1 *µ*l of cDNA, and 1 *µ*l of each primer. PCR amplification was performed using the following conditions: 10 s at 95°C and 45 cycles of 15 s at 95°C and 60 s at 60°C. The relative mRNA expression level was analyzed using the 2^−ΔΔCT^ calculation method. The glyceraldehyde-3-phosphate dehydrogenase (GAPDH) mRNA expression level was used as an endogenous control. The forward primers and reverse primers were obtained from Sangon Biotech (Shanghai, China), and their respective sequences were as follows: ARHGEF10L: 5′AGTGCCAGGTGGTGTTCTTC3′ and 5′AAGAGGTCCCCGATCTTCTC3'; GAPDH: 5′CAGAACATCATCCCTGCCTCTAC3′ and 5′TTGAAGTCAGAGGAG ACCACCTG3'; HSPA6: 5′ ACTTTCACCACCTACTCGGA 3′ and 5′ CCCTCTCA CCCTCATACAC3'. All experiments were repeated at least three times.

### 2.4. Western Blot Analysis

After transfection, HeLa cells were lysed on ice for 30 min and then centrifuged at 12,000 g for 30 min at 4°C to collect the supernatant. The protein concentrations were determined using a bicinchoninic acid (BCA) protein assay kit (Solarbio, China). Protein samples (50 *µ*g) were transferred to polyvinylidene fluoride membranes (PVDF, Millipore, USA) after 10%–12% sodium dodecyl sulfate polyacrylamide gel electrophoresis (SDS-PAGE). The membrane was incubated with primary antibodies at 4°C overnight. After incubation with the horseradish peroxidase- (HRP-) conjugated secondary antibody, hybridization was detected with Western Chemiluminescent HRP Substrate (Millipore, USA). The ARHGEF10L antibody was commercially obtained from Sigma (catalog number: HPA026426, St. Louis, USA), and GAPDH (catalog number: 10494-1-AP), N-cadherin (catalog number: 22018-1-AP), E-cadherin (catalog number: 20874-1-AP), and HSPA6 (catalog number: 13616-1-AP) antibodies were obtained from Proteintech (USA); ROCK1 (catalog number: 4035), Slug (catalog number: 9585), ezrin/radixin/moesin, and phospho-ezrin (Thr567)/radixin (Thr564)/moesin (Thr558) antibodies (catalog number: 3142 and 3141) were obtained from Cell Signaling Technology (St. Louis, USA), and Bcl-2 (catalog number: BA0412) and Bax (catalog number: BA0315) antibodies were obtained from Boster (China). The ezrin/radixin/moesin antibody included ezrin/radixin (80 kDa) and moesin (75 kDa). All experiments were repeated at least three times.

### 2.5. CCK-8 Assays of Cell Proliferation

To assess cell proliferation, cell counting kit-8 assays (CCK-8, Dojindo, Japan) were performed according to the manufacturer's protocol. Transfected HeLa cells (5 × 10^3^ cells per well) were seeded into 96-well plates and cultured for 72 h. CCK-8 solution (10 *µ*l) was added and incubated for 1–3 h at 37°C. The OD value was determined at an absorbance wavelength of 450 nm using a spectrophotometer (SpectraMax 190, Molecular Devices). All experiments were repeated at least three times.

### 2.6. Wound-Healing Assay of Cell Migration

A wound-healing assay was performed to assess cell migration. HeLa cells (10 × 10^5^) were cultured on a six-well plate for 24 h after transfection, and a wound was scratched through the cells using a micropipette tip. The cells were cultured for 24–48 h. Images of cell migration were obtained under a light microscope. All experiments were repeated at least three times.

### 2.7. Hoechst Staining of Cell Apoptosis

Hoechst staining was performed to evaluate apoptosis in the transfected cells. HeLa cells were seeded in 24-well plates on sterile cover glasses. A total of 5 × 10^5^ cells were fixed with 0.5 ml of fixing solution for 20 min and washed twice with PBS. The cells were stained with 0.5 ml Hoechst 33258 from a Hoechst staining kit (Beyotime Biotechnology, China) for 5 min at room temperature and washed twice with PBS according to the manufacturer's protocol. The cells were immediately imaged using a fluorescence microscope (Olympus FSX100). All experiments were repeated at least three times.

### 2.8. Rho Activation Assay

The Rho Activation Assay kit (Cytoskeleton, Denver, USA) was used according to the manufacturer's instructions to assess the activity of Rho GTPases. After transfection, 5 × 10^6^ HeLa cells were washed twice in ice-cold PBS and harvested with ice-cold cell lysis buffer supplemented with 1 × protease inhibitor cocktail. Then, the lysates were immediately clarified by centrifugation at 10,000 g at 4°C for 1 min, after which the cell supernatant was incubated with 15 *µ*l rhotekin-RBD beads for 1 h at 4°C using gentle rotation. Total protein from HeLa cells was mixed with a GST-tagged fusion protein corresponding to residues 7–89 of rhotekin. The beads were washed with cold washing buffer and collected by centrifugation. GTP-Rho complexed with rhotekin-RBD beads was pulled down. Then, the beads were resuspended in 2 × Laemmli buffer and boiled. Western blot analysis was performed with a specific RhoA antibody in the kit. The GTP-Rho proteins were immunoblotted with an anti-RhoA antibody in the kit. The total protein from the cells that were transfected with the blank plasmids was used as a control. All experiments were repeated at least three times. We performed this experiment in our previous study [8].

### 2.9. RNA Sequencing Assay

HeLa cells were transfected with ARHGEF10L-expressing plasmids or mock expression vectors by using PolyJet^TM^ DNA In Vitro Transfection Reagent according to the manufacturer's protocol. The 6 × 10^5^ transfected cells were washed with cold PBS and collected in TRIzol (Invitrogen™, USA). RNA sequencing was performed by the OE Biotech Company (Shanghai, China). The libraries were constructed using the TruSeq Stranded mRNA LT Sample Prep Kit (Illumina, San Diego, CA, USA). These libraries were generated and sequenced on the Illumina sequencing platform (HiSeqTM 2500 platform). Kyoto Encyclopedia of Genes and Genomes (KEGG) and gene ontology (GO) pathway analyses were used to predict the functions of the differentially expressed mRNAs. Differentially expressed mRNAs were defined as mRNAs with a fold change >2 and a *P* value < 0.05. Each group comprised three duplicate samples.

### 2.10. Statistical Analysis

We performed a statistical analysis with collective data from three or more replicates of each experiment. Student's *t*-test was applied to analyze the statistical significance using SPSS software. Data are expressed as the mean ± standard deviation (SD). *P* < 0.05 was considered statistically significant.

## 3. Results

### 3.1. Effect of ARHGEF10L Expression on the Proliferation, Migration, and Apoptosis of HeLa Cells

**Figure 1:**
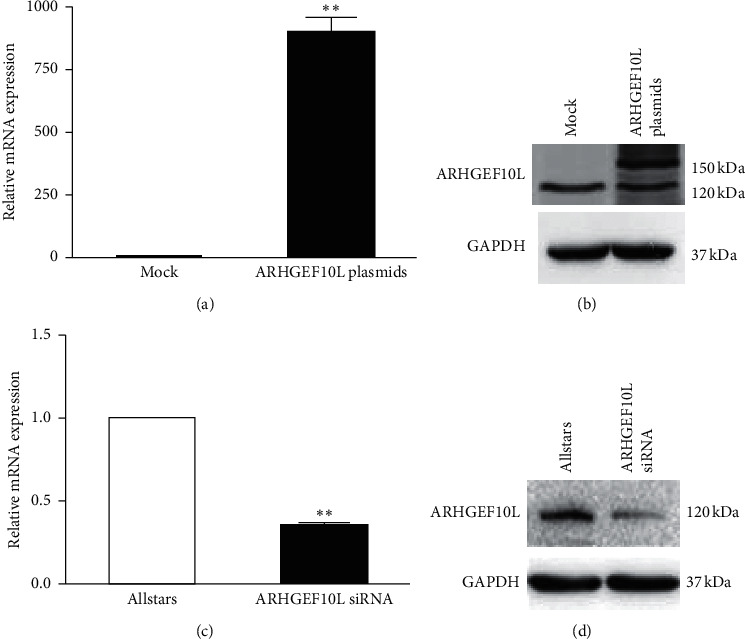
ARHGEF10L expression in HeLa cells. (a) Quantitative real-time PCR and (b) Western blotting detected the ARHGEF10L expression level in HeLa cells transfected with ARHGEF10L-expressing plasmids. (c) Quantitative real-time PCR and (d) Western blotting detected the ARHGEF10L expression level in HeLa cells transfected with anti-ARHGEF10L siRNA. ^*∗∗*^*P* < 0.01.

The ARHGEF10L-expressing plasmids and the mock expression vectors were transfected into HeLa cells. The expression of exogenous ARHGEF10L was detected by qRT-PCR and Western blotting. The qRT-PCR results showed increased mRNA levels of ARHGEF10L in the transfected HeLa cells compared with the cells transfected with blank expression plasmids (mock) (Figure 1(a)). The Western blotting results showed increased protein levels of ARHGEF10L in the transfected HeLa cells compared with the mock-transfected cells (Figure 1(b)). Since recombinant ARHGEF10L protein with an RFP tag was overexpressed, the molecular weight of the immune signal in the transfected cells was higher than that in the mock sample without the RFP tag. At the same time, anti-ARHGEF10L and AllStars siRNA were transfected into HeLa cells. qRT-PCR results showed decreased mRNA levels of ARHGEF10L in the cells that were transfected with AllStars siRNA (Figure 1(c)). Western blotting showed decreased protein levels of ARHGEF10L in the siRNA-transfected cells compared with the control cells (Figure 1(d)).

**Figure 2:**
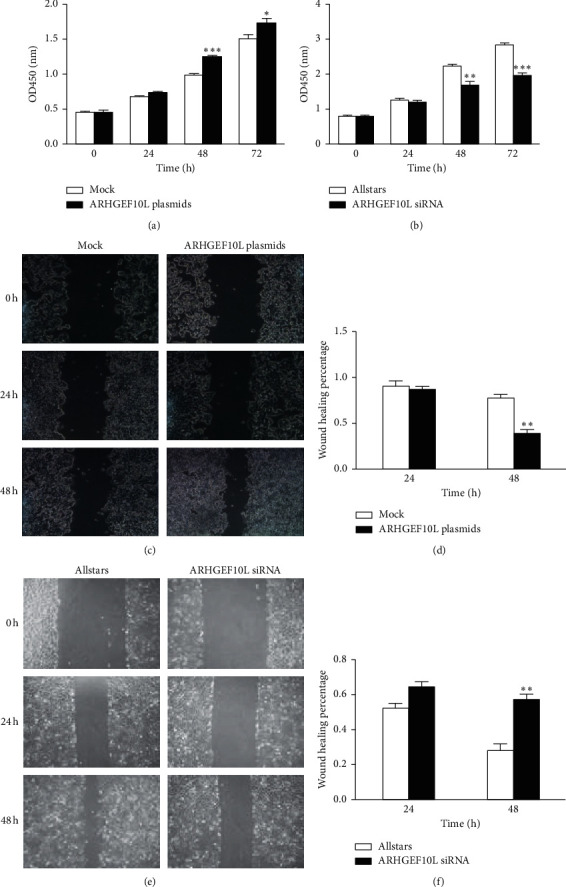
Effects of ARHGEF10L expression on cell proliferation and migration. (a) Cell proliferation was detected in HeLa cells transfected with ARHGEF10L-expressing plasmids using CCK-8 assays. (b) Cell proliferation was detected in HeLa cells transfected with anti-ARHGEF10L siRNA using CCK-8 assays. (c) Cell migration was detected in HeLa cells transfected with ARHGEF10L-expressing plasmids using wound-healing assays. (d) Quantitative analysis of HeLa cell migration following plasmid transfection. (e) Cell migration was detected in HeLa cells transfected with anti-ARHGEF10L siRNA using wound-healing assays. (f) Quantitative analysis of HeLa cell migration following siRNA transfection. ^*∗*^*P* < 0.05, ^*∗∗*^*P* < 0.01, and ^*∗∗∗*^*P* < 0.001.

CCK-8 assays were used to determine the effect of ARHGEF10L expression on cell proliferation. Compared to HeLa cells transfected with blank expression vectors (mock), HeLa cells transfected with the ARHGEF10L-expressing plasmids showed significantly increased cell proliferation following transfection for 48 h and 72 h (Figure 2(a)). In contrast, decreased cell proliferation was detected in HeLa cells transfected with anti-ARHGEF10L siRNA for 48 h and 72 h compared with cells that were transfected with AllStars siRNA (Figure 2(b)). Because the wound-healing assay did not detect a significant change in migration at 72 h after scratching, we did not show the migration data at 72 h.

The effect of ARHGEF10L expression on cell migration was determined using wound-healing assays. Compared to HeLa cells transfected with the blank expression vectors, HeLa cells transfected with ARHGEF10L-expressing plasmids showed increased migration ability following transfection for 48 h (Figures 2(c) and 2(d)). HeLa cells transfected with anti-ARHGEF10L siRNA showed decreased migration ability compared with cells transfected with AllStars siRNA (Figures 2(e) and 2(f)).

**Figure 3:**
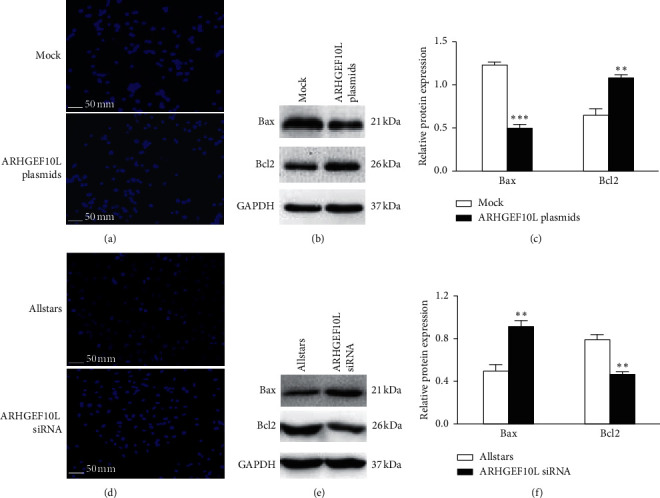
Effects of ARHGEF10L expression on apoptosis in HeLa cells. (a) Hoechst staining showed apoptosis in HeLa cells transfected with ARHGEF10L-expressing plasmids. (b) Bcl-2 and Bax protein expression in HeLa cells transfected with ARHGEF10L-expressing plasmids was detected using Western blotting. (c) Semiquantitation of the protein expression of Bcl-2 and Bax in HeLa cells. (d) Hoechst staining showed apoptosis in HeLa cells transfected with anti-ARHGEF10L siRNA. (e) Bcl-2 and Bax protein expression in HeLa cells transfected with anti-ARHGEF10L siRNA was shown using Western blotting. (f) Semiquantitation of the protein expression of Bcl-2 and Bax in HeLa cells. ^*∗∗*^*P* < 0.01 and ^*∗∗∗*^*P* < 0.001.

The effect of ARHGEF10L expression on cell apoptosis was detected by Hoechst staining assays and by measuring the protein expression of Bcl-2 and Bax using Western blotting. Compared with that in the control cells transfected with blank expression vectors (mock), a decrease in the fluorescence signals of Hoechst 33258 was observed in HeLa cells transfected with the ARHGEF10L plasmids (Figure 3(a)), which indicates a low number of apoptotic cells following transfection of the expressing plasmids. Bcl-2, an antiapoptotic protein, showed increased expression. Bax, a proapoptotic protein, showed decreased expression in ARHGEF10L-expressing HeLa cells (Figures 3(b) and 3(c)). Following the silencing of ARHGEF10L expression with siRNA, HeLa cells showed increased fluorescence signals of Hoechst 33258 compared with the cells transfected with AllStars siRNA (Figure 3(d)), which indicates a high number of apoptotic cells following siRNA transfection. Bcl-2 showed decreased expression, and Bax showed increased expression in ARHGEF10L-silenced HeLa cells (Figures 3(e) and 3(f)).

The above results indicated that ARHGEF10L overexpression elevated migration and proliferation and reduced apoptosis in HeLa cells. In contrast, silencing ARHGEF10L expression in HeLa cells suppressed migration and proliferation and promoted apoptosis in HeLa cells.

### 3.2. Effect of ARHGEF10L Overexpression on Rho Pathway Activation in HeLa Cells

**Figure 4:**
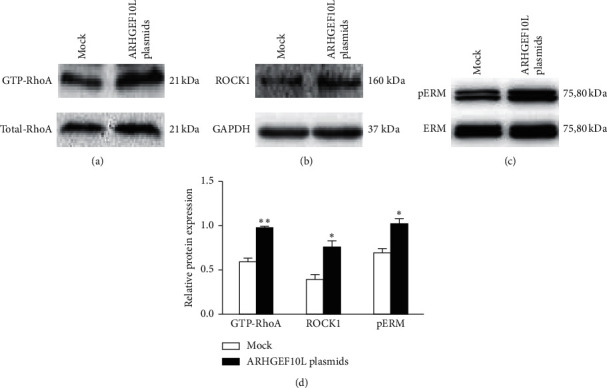
Effects of ARHGEF10L overexpression on the RhoA pathway in HeLa cells. (a) Western blotting revealed the protein expression of GTP-RhoA in HeLa cells transfected with ARHGEF10L-expressing plasmids or blank vectors (Mock). (b) Western blotting revealed the protein expression of ROCK1 in HeLa cells transfected with the ARHGEF10L-expressing plasmid or the mock plasmid. (c) Western blotting revealed the protein expression of phospho-ezrin/radixin/moesin (pERM) in HeLa cells transfected with ARHGEF10L-expressing plasmids or mock. (d) Semiquantitation of the protein expression of GTP-RhoA, ROCK1, and pERM in HeLa cells. ^*∗*^*P* < 0.05 and ^*∗∗*^*P* < 0.01.

We used the Rho activation assay to measure the effects of ARHGEF10L on GTP/GDP-Rho GTPases in HeLa cells. Total protein from HeLa cells was mixed with a GST-tagged fusion protein corresponding to residues 7–89 of rhotekin. GTP-Rho complexed with rhotekin-RBD beads was pulled down and immunoblotted with an anti-RhoA antibody. Compared with the cells that were transfected with blank plasmids, the immunosignals of activated GTP-RhoA were significantly increased in the cells overexpressing ARHGEF10L (Figure 4(a)). This result indicates that recombinant ARHGEF10L protein in HeLa cells possesses GEF activity and can specifically activate GTP-RhoA.

We also investigated the protein expression of ROCK1 and phospho-ERM (phospho-ezrin/radixin/moesin), two other key effectors in the Rho pathway. Western blot analysis detected the increased expression of ROCK1 and phospho-ERM in HeLa cells overexpressing ARHGEF10L (Figures 4(b)–4(d)). The above results suggested that overexpression of ARHGEF10L activated the GTP-RhoA-ROCK1-pERM pathway in HeLa cells.

### 3.3. Effect of ARHGEF10L Overexpression on EMT in HeLa Cells

**Figure 5:**
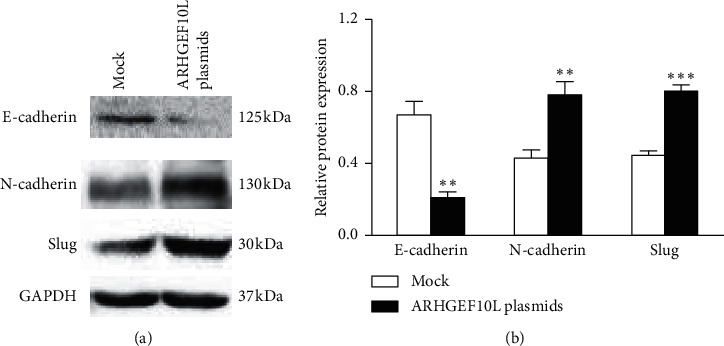
Effects of ARHGEF10L overexpression on EMT in HeLa cells. (a) Expression of the EMT markers E-cadherin, N-cadherin, and Slug in HeLa cells was detected using Western blotting. (b) Semiquantitation of the protein expression of E-cadherin, N-cadherin, and Slug. ^*∗∗*^*P* < 0.01 and ^*∗∗∗*^*P* < 0.001.

Since EMT is the primary process involved in cancer invasion and metastasis, we examined the important role of ARHGEF10L in EMT. We investigated the expression of E-cadherin, N-cadherin, and Slug, three important markers of EMT, in HeLa cells overexpressing ARHGEF10L. Western blotting detected increased expression of N-cadherin and Slug and decreased expression of the epithelial marker E-cadherin in HeLa cells overexpressing ARHGEF10L (Figures 5(a) and 5(b)). The results indicated that the upregulation of ARHGEF10L resulted in the induction of EMT in HeLa cells.

### 3.4. RNA Sequencing Analysis of ARHGEF10L-Overexpressing HeLa Cells

RNA sequencing assays were performed in HeLa cells transfected with ARHGEF10L-expressing plasmids or mock plasmids. Differentially expressed mRNAs with a fold change >2 and *P* < 0.05 were identified. Of all the differentially expressed genes identified, the mRNA expression levels of 22 genes were upregulated, and those of 9 genes were downregulated. The results are shown in Figure Supplement 1. Kyoto Encyclopedia of Genes and Genomes (KEGG) and gene ontology (GO) pathway analyses were used to predict the functions of the differentially expressed mRNAs, and the results are presented in Figure Supplement 2. The analyses identified the pathways showing significant differences, including cyclin-dependent protein serine/threonine kinase activity, cell response to vitamin A, and Toll-like receptor signaling pathways.

**Figure 6:**
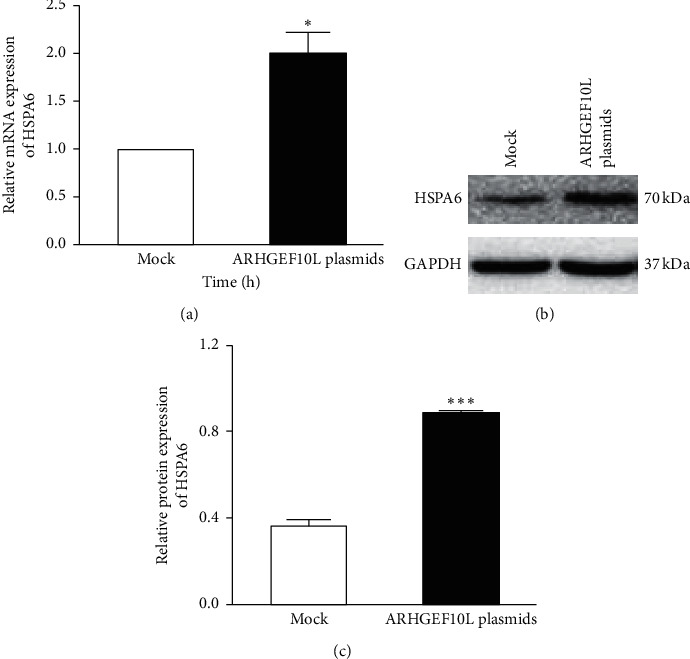
Effects of ARHGEF10L overexpression on HSPA6 expression in HeLa cells. (a) The mRNA expression of HSPA6 was detected using real-time PCR in HeLa cells transfected with ARHGEF10L-expressing plasmids or blank vectors (Mock). (b) The protein expression of HSPA6 was detected using Western blotting in HeLa cells transfected with the ARHGEF10L-expressing plasmid or the mock plasmid. (c) Semiquantitation of the protein expression of HSPA6. ^*∗*^*P* < 0.05 and ^*∗∗∗*^*P* < 0.001.

To confirm the differential expression of genes, we performed qRT-PCR and Western blotting analyses to verify the expression of heat shock 70 kDa protein 6 (HSPA6) because our RNA-sequencing assay also detected an increased expression of HSPA6 in SGC7901 cells overexpressing ARHGEF10L [9]. The qRT-PCR results showed an increase in the mRNA expression of HSPA6 in the cells overexpressing ARHGEF10L compared with the cells transfected with blank plasmids (Figure 6(a)). Western blotting also detected increased protein expression of HSPA6 in the cells overexpressing ARHGEF10L (Figures 6(b) and 6(c)). The present study did not verify any significant differential expression of the other 30 genes that were determined to have alternative expression by RNA sequencing analysis.

## 4. Discussion

In the present study, we investigated cell proliferation using CCK-8 assays, cell migration using wound-healing assays, and cell apoptosis using Hoechst staining assays in HeLa cells. These results showed that ARHGEF10L overexpression promoted cell proliferation, enhanced cell migration, and suppressed cell apoptosis in HeLa cells. Meanwhile, the protein level of Bcl-2 was upregulated, and the protein level of Bax was downregulated. The Bcl-2 protein family, which includes both antiapoptotic proteins (such as Bcl-2, Bcl-xl, and Bcl-w) and proapoptotic proteins (such as Bax, Bak and Bad), regulates all major types of cell death, including apoptosis, necrosis, and autophagy [10–12]. The above results suggest that ARHGEF10L expression stimulated cervical tumorigenesis by promoting cell proliferation and migration and inhibiting cell apoptosis.

We determined the role of ARHGEF10L during EMT in cervical tumors. EMT is a process of cell dedifferentiation involving the adaptation of cellular morphology and behavior. Activating EMT could promote tumor metastasis by inducing cell migration and invasion [13]. During EMT progression, the downregulation of E-cadherin is balanced by the increased expression of N-cadherin, resulting in a “cadherin switch” that alters cell adhesion [14, 15]. Many transcription factors play important roles in the regulation of EMT, such as the zinc finger proteins SNAI1 (Snail) and SNAI2 (Slug), zinc finger E-box-binding homeobox 1 and 2 (ZEB1 and ZEB2), and Twist-related proteins 1 and 2 (TWIST1 and TWIST2). These factors activate specific molecular programs that repress the expression of epithelial markers (E-cadherin) and activate the expression of mesenchymal markers (vimentin) [16]. The RhoA-ROCK pathway has been found to promote cell migration and EMT in many tumors [17–19]. In this study, we found that the overexpression of ARHGEF10L upregulated the expression of the mesenchymal markers N-cadherin and Slug and downregulated the expression of the epithelial marker E-cadherin, which indicates that ARHGEF10L expression stimulated cervical tumorigenesis by promoting EMT.

The Rho-associated coiled-coil protein kinase (ROCK) family, including ROCK1 and ROCK2, has been shown to play a central role in promoting epithelial tumor growth and progression [20, 21]. RhoA acts upon Rho-associated protein kinase (ROCK) [22]. ERM family proteins are thought to function as general cross-linkers between the plasma membrane and actin filaments, and the phosphorylation of ERM is mainly mediated by ROCK, which has been reported to play a critical role in mediating the effects of RhoA [23–25]. Rho and ROCK-dependent ERM phosphorylation regulates Fas-mediated apoptosis in Jurkat cells [26]. Ezrin phosphorylation regulates the invasion and metastasis of breast cancer cells [27] and pancreatic cancer cells [28], and radixin regulates cell migration and cell-cell adhesion in prostate cancer [29]. During EMT, actin filament remodeling depends on the increased expression of the ERM protein moesin [30]; moesin, as an EMT marker, supports the association of moesin, Snail, and EMT and thereby affects the prognosis of breast cancer [31]. In the present study, we detected activation of GTP-RhoA in HeLa cells overexpressing ARHGEF10L using a Rho activation assay. We also detected increased protein expression of ROCK1 and pERM in transfected HeLa cells. The above results support the finding that ARHGEF10L activates the GTP-RhoA-ROCK-pERM pathway in HeLa cells to activate tumor cells during tumorigenesis.

In this study, we assessed the RNA sequence expression profile in ARHGEF10L-overexpressing HeLa cells. We detected changes in the transcriptional expression of 31 genes in the transfected cells, including increased transcription of HSPA6. Compared with the mock-transfected cells, differentially expressed mRNAs (fold change >2, *P* < 0.05) were detected in the cells overexpressing ARHGEF10 L. Among those mRNAs, 22 genes were significantly upregulated and 9 genes were downregulated. We investigated the downstream pathway of ARHGEF10L by RNA sequencing analysis in SGC7901 cells that originated from gastric tumors. Interestingly, the analysis also detected an increased expression of HSPA6 in that study [9]. It is highly probable that HSPA6 is a key factor in the ARHGEF10L downstream pathway. Thus, in the present study, we used Western blot and real-time PCR analyses to verify the increased transcription and translation of HSPA6. We planned to examine the differential expression of the other 30 genes in a future study.

HSPA6, a member of the large HSP70 family, was significantly upregulated and has been targeted as a biomarker of cellular stress. The HSP70 family of heat shock proteins consists of molecular chaperones of approximately 70 kDa in size, which are thought to serve as a potent buffering system against cellular stress caused by physiological, viral, environmental, replication-induced, or oncogenic stimuli [32, 33]. HSP70 overexpression is a marker of undifferentiated ovarian cancer [34] and was found to be correlated with increased proliferation and tumor size in uterine cervical cancer and colorectal carcinoma [35, 36]. Overexpression of the HSPA6 gene was related to the proliferation, migration, and invasiveness of bladder cancer cells [37]. Based on the studies of others, we considered that detection of increased expression of HSPA6 is reasonable in ARHGEF10L-overexpressing HeLa cells. Most likely, ARHGEF10L overexpression promoted tumor cell proliferation, migration, and invasiveness to stimulate HSPA6 expression, although the exact function of HSPA6 in tumorigenesis is not well known. HSPA6 is considered an important tumor-related gene under the control of ARHGEF10L.

RNA sequencing analysis also identified significant changes in cyclin-dependent protein serine/threonine kinase activity, cell response to vitamin A, and Toll-like receptor signaling pathways using KEGG and GO pathway analyses in HeLa cells overexpressing ARHGEF10L. Cyclins and cyclin-dependent protein kinases (CDKs) are important proteins that are required for the regulation and expression of a large number of components necessary for cell cycle progression. Increased cyclin or CDK expression or decreased levels of endogenous CDK modulators/inhibitors have been observed in a wide variety of tumors. Thus, CDKs represent natural targets for anticancer therapies [38]. All-trans retinoic acid (ATRA) is an active metabolite of vitamin A within the retinoid family. Retinoids, through their cognate nuclear receptors, exert potent effects on cell growth, differentiation, and apoptosis and have significant promise for cancer therapy and chemoprevention. ATRA has been increasingly included in antitumor therapeutic schemes for the treatment of various tumor types [39]. Toll-like receptors (TLRs), which are mainly expressed by innate immune cells, are involved in inducing and regulating adaptive immune responses. The expression of TLRs has been detected in many tumors, and stimulation of these receptors results in tumor progression or regression, depending on the TLR and tumor type [40].

We reported that ARHGEF10L played a role through RhoA-ROCK1-ERM signaling, an important pathway in tumorigenesis, and stimulated EMT in liver tumors and gastric tumor cells [8, 9]. We also found that ARHGEF10L expression stimulated HSPA6 expression in SGC7901 cells that were derived from gastric tumors [9]. The current study and a study with gastric tumor cells used RNA sequencing to investigate the downstream mechanism of ARHGEF10L and found increased HSPA6 expression in ARHGEF10L-expressing HeLa cells. Combined with the results of the present study, we suggest that ARHGEF10L plays a tumorigenic role through the RhoA-ROCK1-ERM pathway, activates EMT, and elevates HSPA6 expression in many tumors. ARHGEF10L is novel oncogene in many tumors.

The mechanism of human papillomavirus (HPV) chronic infection resulting in cervical tumors has been recognized. Thus far, we do not understand the relationship between ARHGEF10L expression and HPV infection. Most likely, HPV infection stimulates ARHGEF10L expression during carcinogenesis. The current study detected increased expression of HSPA6, and HSPA6 was detected in hepatoma carcinoma cells. Upregulation of this heat shock protein was associated with poor outcomes in hepatitis B virus- (HBV-) related early-stage hepatocellular carcinoma [41, 42].

## 5. Conclusions

The present study demonstrated that the increased expression of ARHGEF10L promotes cervical cell proliferation and migration and suppresses cell apoptosis. This study also found that ARHGEF10L expression stimulates GTP-RhoA, ROCK1, and phospho-ERM expression and induces EMT in HeLa cells. Additionally, our study detected the increased expression of HSPA6 in the cells that overexpressed ARHGEF10L. These results provide important data to support the crucial role of ARHGEF10L in the tumorigenesis of cervical carcinoma. ARHGEF10L is thus a novel tumor-related gene that plays an important role in tumorigenesis.

## Data Availability

The data used and/or investigated during the present study are available from the corresponding author upon reasonable request.

## Supplementary Materials

Supplementary Materials. Figure Supplement 1: Analysis of the RNA expression profiles in ARHGEF10L-overexpressing HeLa cells. (a) Hierarchical clustering analysis of differentially expressed mRNAs. (b) List of the regulated genes. Sample HR represents the control group, and sample HA represents the experimental group. Figure Supplement 2: KEGG and GO pathway analyses of HeLa cells overexpressing ARHGEF10L. (a) Kyoto Encyclopedia of Genes and Genomes pathway analysis of differentially expressed mRNAs in HeLa cells overexpressing ARHGEF10L. (b) Gene ontology annotations of differentially expressed mRNAs in ARHGEF10L-overexpressing HeLa cells.
