# Supplementary material for: ARHGEF10L Promotes Cervical Tumorigenesis via RhoA-Mediated Signaling
Source: Evid Based Complement Alternat Med. 2021 Mar 24;2021:6683264. doi: 10.1155/2021/6683264 (PMC8012150; doi:10.1155/2021/6683264)
Supplement: Supplementary Materials. — Figure Supplement 1: Analysis of the RNA expression profiles in ARHGEF10L-overexpressing HeLa cells. (a) Hierarchical clustering analysis of differentially expressed mRNAs. (b) List of the regulated genes. Sample HR represents the control group, and sample HA represents the experimental group. Figure Supplement 2: KEGG and GO pathway analyses of HeLa cells overexpressing ARHGEF10L. (a) Kyoto Encyclopedia of Genes and Genomes pathway analysis of differentially expressed mRNAs in HeLa cells overexpressing ARHGEF10L. (b) Gene ontology annotations of differentially expressed mRNAs in ARHGEF10L-overexpressing HeLa cells. [file 6683264.f1.zip › 6683264.f1/Figure Supplement 1.pdf]

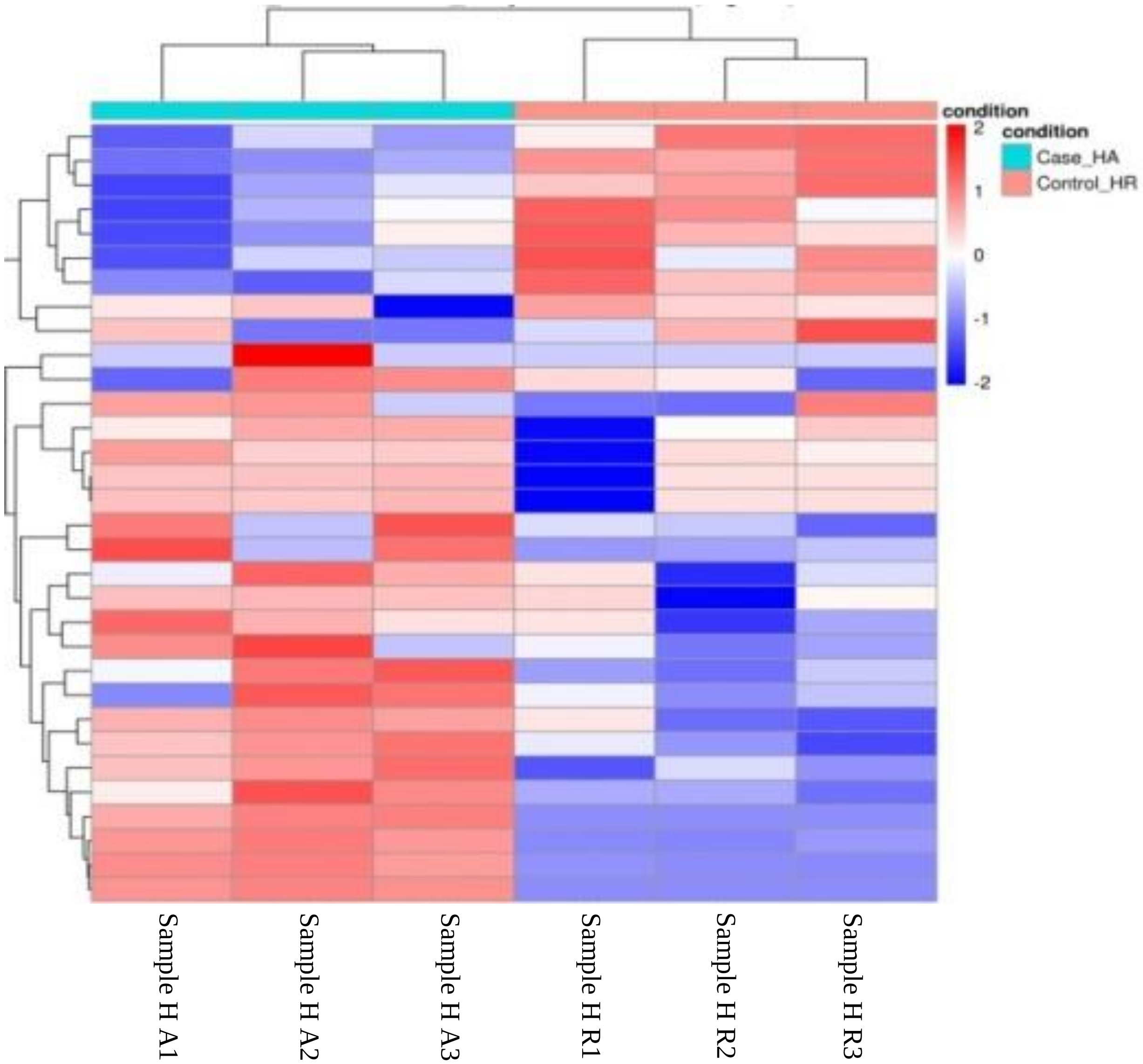

(a)

Differentially expressed mRNAs

GENE	UP_DOWN	GENE	UP_DOWN
ABHD16A	Up	C6orf99	Down
BDP1	Up	CDKN1C	Down
C3orf20	Up	CLK2	Down
CUBN	Up	DYSF	Down
DHRSX	Up	FSIP1	Down
FERMT3	Up	GNL1	Down
FLJ45513	Up	LOC102724235	Down
GTF2H4	Up	PARN	Down
HSPA6	Up	TMEM238	Down
HYOU1	Up		
IFNB1	Up		
IRF7	Up		
KLRG2	Up		
LRIT3	Up		
LTC4S	Up		
PSG5	Up		
SMN2	Up		
SPATC1L	Up		
TMEM50B	Up		
VAMP5	Up		
VWF	Up		
ZNF100	Up		

(b)
