# Supplementary figures and images for: ARHGEF10L Promotes Cervical Tumorigenesis via RhoA-Mediated Signaling
Source: Evid Based Complement Alternat Med. 2021 Mar 24;2021:6683264. doi: 10.1155/2021/6683264 (PMC8012150; doi:10.1155/2021/6683264)

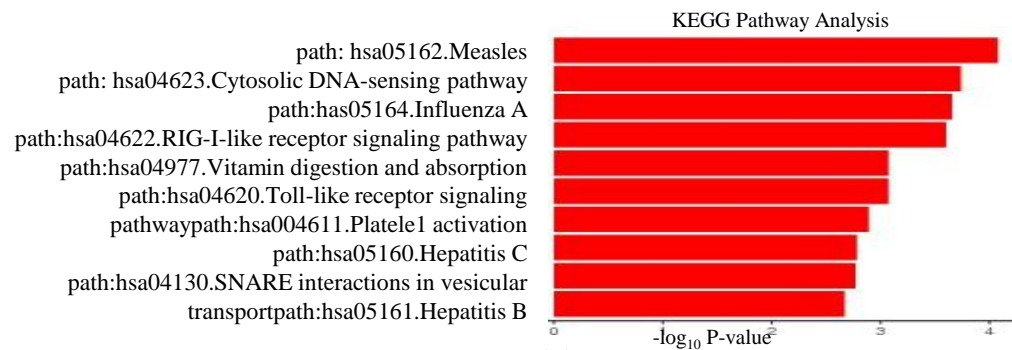

(a)

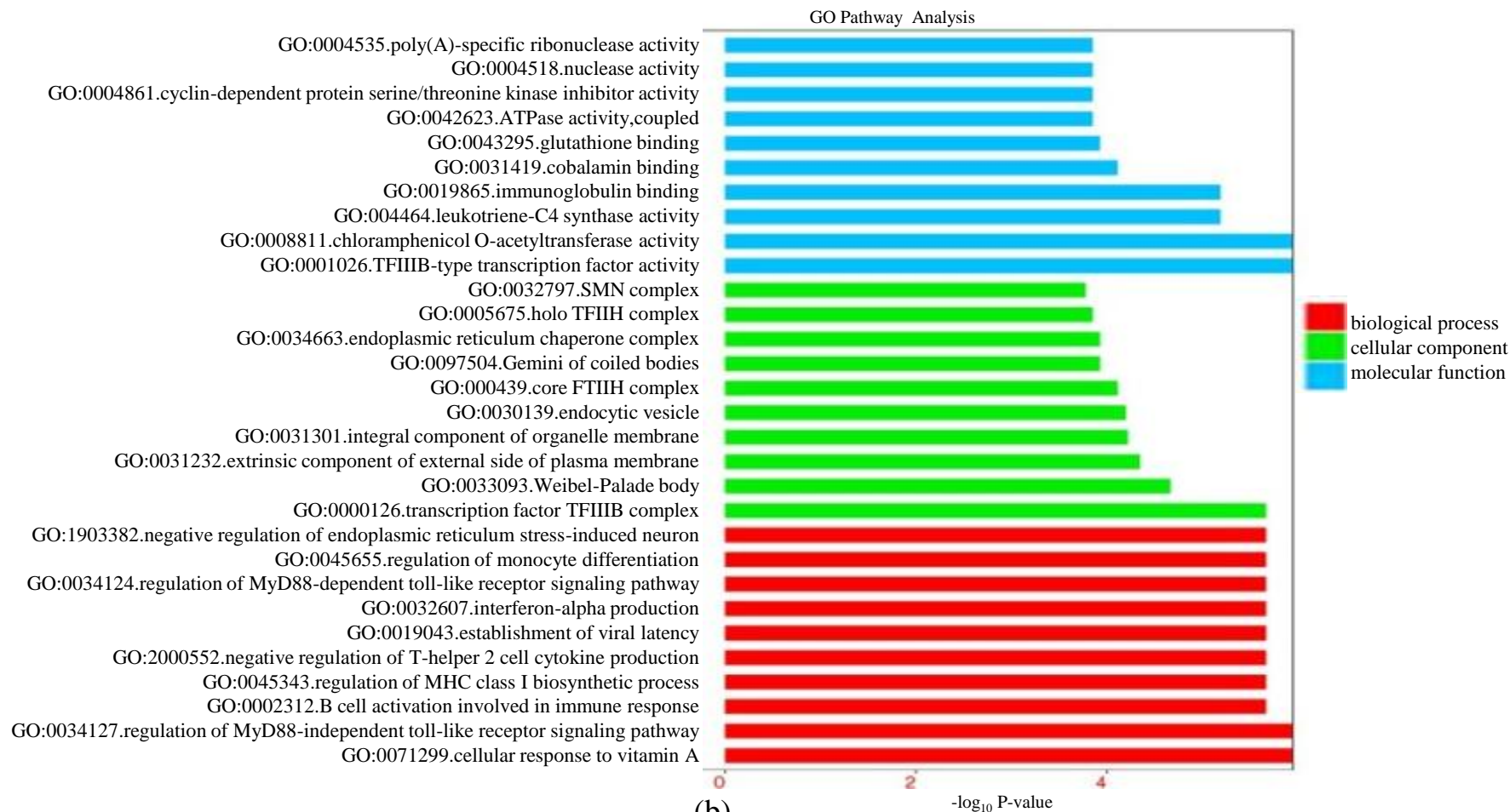

(b)

Supplement: Supplementary Materials. — Figure Supplement 1: Analysis of the RNA expression profiles in ARHGEF10L-overexpressing HeLa cells. (a) Hierarchical clustering analysis of differentially expressed mRNAs. (b) List of the regulated genes. Sample HR represents the control group, and sample HA represents the experimental group. Figure Supplement 2: KEGG and GO pathway analyses of HeLa cells overexpressing ARHGEF10L. (a) Kyoto Encyclopedia of Genes and Genomes pathway analysis of differentially expressed mRNAs in HeLa cells overexpressing ARHGEF10L. (b) Gene ontology annotations of differentially expressed mRNAs in ARHGEF10L-overexpressing HeLa cells. [file 6683264.f1.zip › 6683264.f1/Figure Supplement 2.pdf]
